# Factors Associated With the Adoption of a Patient Education Intervention Among First Responders, King County, Washington, 2010–2011

**DOI:** 10.5888/pcd11.130221

**Published:** 2014-01-30

**Authors:** Hendrika Meischke, Benjamin Stubbs, Carol Fahrenbruch, Elizabeth Phelan

**Affiliations:** Author Affiliations: Benjamin Stubbs, Carol Fahrenbruch, Seattle and King County Division of Emergency Medical Services, Seattle, Washington; Elizabeth Phelan, Department of Medicine, University of Washington, Seattle, Washington.

## Abstract

**Introduction:**

This study investigated facilitators and barriers to adoption of an at-scene patient education program by firefighter emergency medical technicians (EMTs) in King County, Washington.

**Methods:**

We consulted providers of emergency medical services (EMS) to develop a patient education pamphlet in the form of a tear-off sheet that could be attached to the EMT medical incident report. The pamphlet included resources for at-scene patient education on high blood pressure, blood glucose, falls, and social services. The program was launched in 29 fire departments in King County, Washington, on January 1, 2010, and a formal evaluation was conducted in late 2011. We developed a survey based on diffusion theory to assess 1) awareness of the pamphlet, 2) evaluation of the pamphlet attributes, 3) encouragement by peers and superiors for handing out the pamphlet, 4) perceived behavioral norms, and 5) demographic variables associated with self-reported adoption of the at-scene patient education program. The survey was completed by 822 (40.1%) of 2,047 firefighter emergency medical technicians. We conducted bivariate and multivariable analyses to assess associations between independent variables and self-reported adoption of the program.

**Results:**

Adoption of the at-scene patient education intervention was significantly associated with positive evaluation of the pamphlet, encouragement from peers and superiors, and perceived behavioral norms. EMS providers reported they were most likely to hand out the pamphlet to patients in private residences who were treated and left at the scene.

**Conclusion:**

Attributes of chronic disease prevention programs and encouragement from peers and supervisors are necessary in diffusion of patient education interventions in the prehospital care setting.

## Introduction

Chronic diseases such as diabetes and hypertension and unintentional injuries such as falls pose a significant burden on society, both in human suffering and financial cost (1–3). Most interventions in chronic disease prevention and treatment and in fall prevention are limited to people seen in a health care provider’s office, a clinic, or a hospital (4–7). Interventions are needed to reach high-risk people in the community who may or may not have such regular sources of care. Every community in the nation has an emergency medical services (EMS) system, and EMS providers see thousands of people each year in response to a 911 call. EMS providers collect information during patient encounters that may be indicators of chronic diseases such as high blood pressure or low blood glucose or injuries related to falls (8). Potential exists for educating high-risk EMS patients about chronic disease control and injury prevention during an EMS encounter (9). However, EMS providers, including emergency medical technicians (EMTs), firefighter EMTs, and paramedics, are acute-care providers whose main responsibility is to stabilize or transport a patient as efficiently and quickly as possible. Thus, public health interventions may not be perceived as compatible with the mission or duties of these providers.

Research shows that patient education materials alone can affect knowledge of chronic disease (10) and effect behavior change, particularly if these materials are part of an interpersonal intervention (11,12). In one study, EMS providers engaged older adults in a brief interpersonal intervention regarding heart attack symptoms during a nonemergency home visit and provided them with patient education materials. Results indicated that the combination of interpersonal intervention and patient education materials had a greater effect on patient behavior than patient education materials alone (11). Because nonemergency home visits are not a cost-effective way of reaching at-risk community residents with chronic disease information, we developed a patient education intervention to be delivered by EMS providers at the scene during an emergency patient encounter.

This article reports on the evaluation of a brief, at-scene patient-education intervention that included delivery of patient education materials by EMTs at part of an EMS response. The objective of our study was to assess the factors that influence adoption of the intervention by firefighter EMTs in fire departments in King County, Washington, a large metropolitan area in the Pacific Northwest. We used the Diffusion of Innovation theory to develop hypotheses regarding firefighters’ adoption of the at-scene intervention (13).

Diffusion theory suggests that an innovation can relate to any product, policy, idea, or practice that is different from the status quo. An at-scene educational intervention would likely be perceived as something new for EMS providers because the focus of their job is on acute patient care rather than education. Diffusion theory further postulates that adoption of an innovation (ie, patient education program) depends on 1) awareness of the program; 2) evaluation of program attributes (is the innovation perceived to be superior to the status quo [relative advantage], compatible with beliefs, costly, difficult or easy to adopt, observable [are others doing it]); and 3) communication with peers or superiors (social influence). One of the assumptions of diffusion theory is that adoption of an innovation is largely under the individual’s control as opposed to adoption of new clinical guidelines, which are mandated by the individual’s superior (13).

### At-scene patient education intervention

We developed an EMT-delivered patient education intervention for community residents who called 911 for a nonlife-threatening event. Patient education focused on blood pressure management (if it was found to be high during the EMS response), blood glucose management (if it was found to be low during the EMS response), or fall prevention (if the 911 call was due to a fall). We pretested the print materials with members of an EMT advisory board consisting of volunteer EMS providers who meet regularly to discuss patient care and EMS involvement in research projects. Rather than create stand-alone materials that were likely be misplaced, the EMT advisory board advised us to develop a patient education pamphlet that could be attached to the back of the medical incident report form (MIRF), which EMS providers are required to complete at the scene. The pamphlet, referred to here as the MIRF backer, included information on high blood pressure, blood glucose, fall prevention, community resources, and telephone numbers of social service agencies and was designed to facilitate a conversation about these topics between EMS providers and patients. The pamphlet, which included personalized medical information obtained during the visit, could be detached from the MIRF and given to the patient (Figure).

**Figure:**
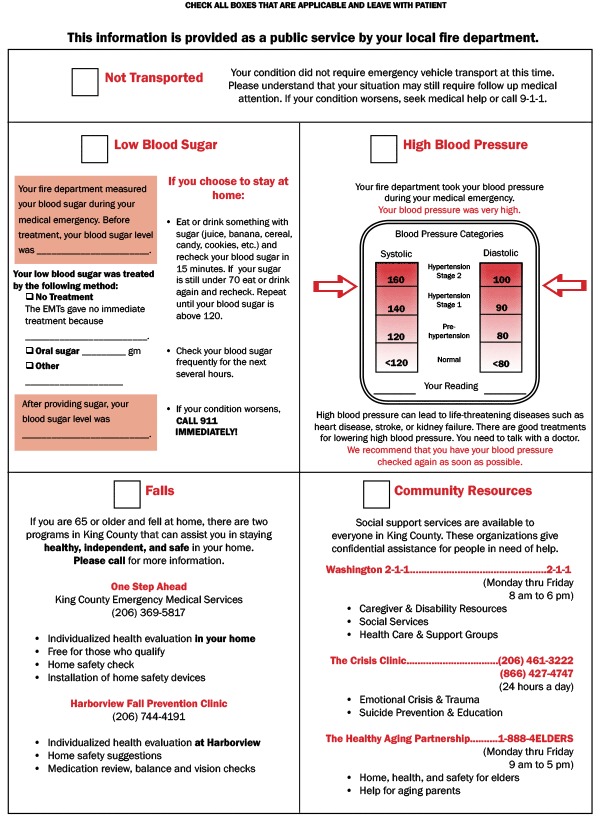
MIRF backer patient education pamphlet.

We hypothesized that adoption of the EMS-based patient education intervention would be greater among EMTs who reported they were aware of the MIRF-backer pamphlet, had more positive perceptions of the pamphlet, and had been encouraged by their peers and superiors to use the pamphlet during EMS patient encounters than among those who had not seen the pamphlet.

## Methods

### Study setting

The EMS patient education project, which we called the “MIRF-backer project” (from the attachment of the patient education pamphlet to the back of the MIRF form), was launched in January 2010 in all 29 fire departments in King County, Washington. These fire departments employed 2,047 certified firefighter EMTs and served more than 936,000 people (14). EMS response in King County, Washington, consists of a 2-tiered system wherein firefighter EMTs are dispatched to the scene immediately to provide basic life support, and paramedics are dispatched if advanced life support is needed. The EMS patient education program was implemented only among providers of basic life support. The medical director of the EMS division of the Seattle King County Public Health Department presented the program during an annual meeting of more than 400 EMS training officers. Training officers could choose, but were not required, to adopt the program and train their EMTs on use of the MIRF-backer. Although use of the MIRF backer was not mandated as a clinical care practice, EMTs were urged to use the pamphlet at scene with appropriate patients, particularly with patients they did not transport to a hospital or clinic.

### Evaluation of the diffusion of the MIRF backer

We developed a brief survey to be administered to firefighter EMTs that was based on the constructs of diffusion theory. Our interest was in identifying barriers to use of the MIRF backer and what facilitated its use with patients who called 911 for a medical emergency. We distributed paper surveys to firefighter trainers in King County, Washington, from December 11, 2011, through February 23, 2012. Trainers then distributed the survey to EMTs. We also made a Web-based survey available for completion from February 13, 2012, through March 21, 2012. Although the survey was designed for 1 EMT to complete, EMTs typically function as part of a 3- or 4-person crew. Thus, we developed survey questions around crew behavior rather than individual behavior.

We measured adoption with 1 primary question, “How often (almost never, sometimes, often, almost always) do you or does someone on your crew hand out the MIRF backer when you have a patient who fits one of the conditions listed on the backer?”

To determine if use of the MIRF backer varied by circumstances of the 911 situation we asked how likely (less likely, no difference, more likely) the EMT was to hand out the MIRF backer in 9 different situations: 1) the patient is left at the scene, 2) the patient is transported, 3) the patient has a life-threatening or other serious condition, 4) the incident prompting the 911 call occurs at a private residence, 5) the incident occurs in a public place, 6) a friend or family member is present, 7) the patient is alone, 8) a language barrier exists, or 9) the incident occurs in a nursing home.

To assess awareness we asked respondents to agree (strongly agree or agree) or disagree (strongly disagree or disagree) with the following statement, “I understand the purpose of the MIRF backer.”

We measured program attributes with 8 statements about the MIRF backer. Responses to some of the innovation attribute statements were highly correlated, so we combined the correlated items into one 5-item scale and treated the other 3 items as separate variables. The 5-item scale consisted of the following statements: 1) “Handing out the MIRF backer gives the patient important health information,” 2) “The MIRF backer is a useful tool to communicate with patients,” 3) “Handing out the MIRF backer is a good opportunity to have a conversation with the patient about his or her health,” 4) “Handing out patient education materials such as the MIRF backer fits our mission to provide prehospital care in our community,” and 5) “Handing out the backer is a good use of EMT time.” Cronbach’s α was 0.89 for this scale. All 5 items address the “relative advantage” concept of innovation attributes in diffusion theory.

Two questions measured specific barriers to adoption: 1) “It’s difficult to remember to hand out the MIRF backer.” 2) “Patients and family or others with them are not receptive to the information contained on the backer.” One item assessed perceived behavioral norms around MIRF-backer use: “In our department most EMTs hand out the MIRF backer.” Responses were scored as 4 (strongly agree), 3 (agree), 2 (disagree), and 1 (strongly disagree); the mean score for each question was calculated. Scores for the 5 relative-advantage questions were combined to calculate an overall score for the relative advantage scale.

We measured communication about the MIRF backer with 1 question that had multiple response options: “Who encouraged you to hand out the MIRF backer?” Response categories were “EMTs,” “training officer,” “fire chief,” “no one,” and “other.”

We asked EMTs how long they had been an EMT in King County (<1 y, 1–9 y, 10–19 y, ≥20y); their age, sex, and race/ethnicity (respondents could indicate more than 1 race); and the name of their fire department.

### Procedures

Surveys were distributed in coordination with supervisors at each fire department responsible for ongoing training of firefighter EMTs. Training supervisors at 25 of the 29 fire departments agreed to distribute paper copies of the survey at continuing education sessions at each department. Four of the 29 fire departments preferred that the survey be completed via the Internet, so the study team created an Internet version of the survey and composed an e-mail message describing the purpose and content of the survey with a link to the survey website. Training supervisors distributed the e-mail message to all their firefighter EMTs, requesting that they complete the survey. This study was reviewed and approved by the University of Washington Human Subjects Division.

### Analysis

We used descriptive statistics to characterize survey respondents and their use of the MIRF backer under various circumstances. We used ANOVA for testing bivariate relationships between the outcome (adoption) and independent variables. An α level of 0.05 was used to determine significance. We used logistic regression to predict EMTs’ frequent use of the MIRF backer (often and almost always) compared with infrequent use of the MIRF backer (almost never) to polarize adoption groups. Standard errors and *P* values were adjusted in the logistic regression analysis to account for clustering by fire department. Analyses were conducted with SPSS Statistics version 19 (IBM Corp, Amonk, New York).

## Results

We received 822 survey responses from 2,047 firefighter EMTs in the county for an overall response rate of 40.1%. Response rates varied by fire department with a mean response rate of 43% (range, 8.0%–90.0%). We received no responses from 2 departments and therefore excluded them from analysis. Response rates were higher in departments that handed out paper versions of the survey than in departments that used the Web-based survey, a mean response rate of 45.0% versus 27.0%.

Survey respondents reflected the demographic characteristics of the King County firefighter EMT population as a whole: largely male (94%), non-Hispanic white (80%), and middle-aged (mean 41 years). Of the 822 responses, 19% reported they almost never handed out the MIRF backer to appropriate patients; 46%, sometimes; 23%, often; and 11%, almost always. Fifty-four percent of respondents reported that they often or almost always were able to discuss the information on the pamphlet when they handed it out.

 Self-reported use of the MIRF backer varied under different circumstances (Table 1). The data show that EMTs were most likely to distribute the pamphlet to patients who were left at the scene. Circumstances in which the MIRF backer was less likely to be used included responses to 911 calls from nursing homes or care centers or in the presence of a language barrier.

**Table 1:** Likelihood of a Firefighter Emergency Medical Technician (N = 831) Using a Patient Education Pamphlet Under Various Patient-Care Circumstances, King County, Washington, 2010–2011

Patient’s Circumstances	Less Likely to Hand Out Pamphlet, %	Does Not Affect Likelihood, %	More Likely to Hand Out Pamphlet, %
Patient left at scene	15	25	59
Patient transported	66	25	9
Patient has serious condition	77	15	9
Incident occurs in private residence	11	54	35
Incident occurs in public place	35	59	7
Incident occurs in nursing home or care center	58	37	6
Friend or family member is present	13	59	28
Patient is alone	15	57	28
Language barrier present	53	37	9

EMTs who reported greater understanding of the MIRF-backer, its relative advantage, behavioral norms, perceived receptivity of the patient or family and friends to the pamphlet, and less difficulty remembering to give the pamphlet to the patient were more likely to report handing out the pamphlet often or almost always than EMTs who did not report regular use of the pamphlet during patient care (Table 2). In addition, encouragement from peers, training officers, and fire chiefs was significantly associated with adoption of the MIRF backer. Lack of encouragement of any kind was significantly negatively associated with handing out the backer. Age and sex were not significantly associated with MIRF-backer use (Table 3). Of the 822 survey respondents, 657 (80%) reported their race as white. Of the 20.0% (n = 164) who did not report their race as white, 73% (n = 119) did not report any race. Because of this high percentage of missing values, we did not include race in our analyses.

**Table 2:** Likelihood of Firefighter EMTs (N = 822) Using a Patient Education Intervention, by Source of Information on the Intervention, and Perception of the Intervention, Based on Self-Report, King County, Washington, 2010–2011

Variable	Almost Never (n = 158)	Sometimes (n = 375)	Often or Always (n = 289)	*P* Value^a^
**Source of Information on the intervention^b^**				
**Peer EMT**				
Yes (n = 152)	1	34	64	<.001
No (n = 670)	23	48	29	
**Training officer**				
Yes (n = 292)	13	39	48	<.001
No (n = 530)	23	50	28	
**Fire chief**				
Yes (n = 46)	2	28	70	<.001
No (n = 776)	20	47	33	
**No one**				
Yes (n = 285)	32	49	19	<.001
No (n = 537)	12	44	35	
**Other**				
Yes (n = 166)	15	51	35	.166
No (n = 656)	20	44	35	
**Perception of the intervention^c^**				
I understand the purpose of the pamphlet (n = 811)	2.9	3.1	3.3	<.001
Relative advantage (n = 766)	2.1	2.5	2.8	
Behavioral norms (n = 782)	1.5	2.0	2.6	
Difficulty remembering to hand out the backer^d^ (n = 809)	1.9	2.0	2.5	
Perceived receptivity of patient or family member to pamphlet’s information (n = 799)	2.0	2.5	2.7	<.001

Abbreviation: EMT, emergency medical technician.

a *P* values were calculated by using χ^2^ and ANOVA statistics.

b Multiple responses possible.

c Responses are based on 5 questions and were scored on a 4-point Likert scale (4 = strongly agree, 3 = agree, 2 = disagree, 1 = strongly disagree). Mean scores are presented here.

d The greater the score, the less difficulty the EMT had in remembering to hand out the MIRF backer and the more receptive the EMT perceived patients and their family members to be. Percentages have been rounded and may not total 100%.

**Table 3:** Logistic Regression Analysis Predicting Firefighter Emergency Medical Technicians’ (N = 342) Adoption of or Failure to Adopt a Patient Education Intervention, King County, Washington, 2010–2011^a^

Firefighter Characteristic	Odds Ratio (95% CI)	*P* Value^b^
Awareness of the pamphlet	1.03 (0.53–1.98)	.927
Perception of relative advantage^c^	3.32 (2.04–5.42)	<.001
Perception of behavioral norms^d^	5.64 (3.12–10.22)	<.001
Difficulty remembering to hand out pamphlet	1.67 (1.25–2.22)	<.001
Patient receptivity	1.51 (0.85–2.67)	.153
**Encouraged by someone to use pamphlet**		
Yes	2.38 (1.32–4.31)	.004
No	1 [Reference]	
**Demographic characteristics**		
Age	0.95 (0.92–1.00)	.051
Sex	2.91 (0.37–22.87)	.310
**Length of service, y**		
<1	0.76 (0.16–3.59)	.73
1–9	0.80 (0.02–2.45)	.70
10–19	0.82 (0.38–1.79)	.63
≥20	1 [Reference]	

a Always or often hands out pamphlet versus almost never hands out pamphlet.

b The Wald test was used to calculate *P* values.

c Relative advantage refers to the belief that the innovation (the patient education intervention) is better than the status quo (not having access to this intervention for at-scene education).

d Behavioral norms refer to the perception that peer EMTs from one’s own department use the MIRF backer for at-scene patient education.

Perceptions of behavioral norms (“most EMTs in my department hand out the MIRF backer”) and relative advantage, remembering to hand out the MIRF backer, and encouragement were significantly associated with self-reported use of the MIRF backer in the multivariate analysis (Table 3).

## Discussion

We explored factors related to the adoption of an at-scene patient education intervention focused on prevention of chronic disease and unintentional injury and delivered by firefighter EMTs. The results show that strong predictors of use of the MIRF backer were perceived benefits (relative advantage) of using the MIRF backer during 911 incidents; encouragement from peers, training officers, and fire chiefs; and perceptions of behavioral norms on use of the MIRF in one’s own fire department.

Although age was not significantly associated with use of the MIRF backer, junior firefighter EMTs reported greater use of the MIRF backer. The fire departments in King County have participated in many other patient outreach programs over the years (11,15,16), and enthusiasm for new programs may wane over time. Alternatively, newly trained first responders may be more open to new approaches to patient care because they might not have firmly established habits. More research is needed to assess whether patient education programs would be more readily adopted if a patient education intervention like ours were part of EMT training of new employees.

Previous research shows that EMS-delivered chronic disease education can affect knowledge and behavior (11,12,16), but monetary costs of such interventions are high. Our study shows that a brief at-scene intervention in combination with patient materials is feasible. EMS providers see so many patients each year that reaching only a small percentage of them with the intervention could have a significant effect on patient health, particularly for community residents who do not have regular health care. Among the patients seen by the 29 fire departments, approximately 35% were not transported to hospital, representing approximately 56,000 patient interactions per year in the study area for whom the 911 encounter may be their only interaction with the health care system. The results of our survey suggest that these patients are perceived as the best targets for an intervention by EMS providers. On a community level, this will translate into a large number of residents receiving patient education at the scene who otherwise might not interact with the health care system.

Our study had limitations. Although the sample size was large, the overall response rate was low and varied dramatically among the fire departments. However, sufficient variation in the outcome measure (use of the MIRF backer) allowed for comparisons of adoption patterns between 2 groups of providers, those who handed out the pamphlet often or always versus those who handed it out almost never. We relied entirely on EMS providers’ self-reported use of the MIRF backer. Identification and timely follow-up with patients who received the MIRF backer was too challenging to be a feasible source for data collection. Future research needs to explore other avenues for confirming at-scene patient education practices.

New patient education initiatives in fire departments should be accompanied by clear and widespread communication of the purpose of the initiative, and EMS providers should be encouraged to adopt the intervention voluntarily. Following diffusion theory principles, it would be interesting to further investigate characteristics of individual fire departments (in addition to individual-level [EMT] characteristics) in relationship to the adoption of patient education interventions, including the presence of an advocate for the program, communication channels and networks, and early-adopter versus late-adopter EMT crews or fire departments.

The MIRF backer was perceived as more useful for patients who were left at scene rather than transported, indicating that the pamphlet was most useful in nonurgent EMT encounters. This group of patients is less likely to receive education than patients who are transported to hospital. As such it is encouraging to note that EMTs perceive this group of patients as appropriate for the intervention. Further investigation needs to be conducted on the effect of such interventions on patient knowledge and behavior.
